# Forsythiaside A Improves the Inhibitory Efficiency of Recombinant Protein Vaccines against Bovine Viral Diarrhea Virus Infection

**DOI:** 10.3390/ijms23169390

**Published:** 2022-08-20

**Authors:** Guanghui Yang, Jiufeng Wang, Shenghua Wang, Yaohong Zhu

**Affiliations:** 1 College of Veterinary Medicine, China Agricultural University, Beijing 100193, China; 2 OIE Porcine Reproductive and Respiratory Syndrome Reference Laboratory, China Animal Disease Control Center, Beijing 102629, China

**Keywords:** BVDV, FTA, cellular immunity, antiviral

## Abstract

Bovine viral diarrhea virus (BVDV) is a critical animal pathogen that leads to cattle production losses associated with acute disease, immune dysregulation, reproductive failure, and respiratory disease. Due to the monotonous control technique and neglect of BVDV, increasing prevalence of BVDV has caused significant economic losses in the cattle industry worldwide. Therefore, novel anti-BVDV drugs are essential to prevent and control BVDV. Our previous studies have found that Forsythoside A (FTA) could inhibit the replication of BVDV via TRAF2-dependent CD28-4-1BB signaling in bovine peripheral blood mononuclear cells (PBMCs), but whether they can directly inhibit the BVDV remains unclear. Here, we further investigated the effects of FTA on BVDV and its underlying mechanisms of action. We found that FTA significantly inhibited the replication of BVDV in the MDBK cell directly. The results demonstrated that FTA could reduce the functional activation of Caspase-1 to inhibit the inflammatory response caused by BVDV infection and increase the expression of type I interferon (IFN-I) to clear the virus in vitro. The animal experiment was performed to evaluate the antiviral effect of FTA in vivo. Notably, after challenged with BVDV, mice with FTA + E^rns^-E2 protein displayed alleviated pathological damage and decreased the viral load in the spleen compared with mice inoculated with E^rns^-E2 protein. Furthermore, treatment with FTA enhanced body defense and delayed infection by the BVDV. Our results reveal that FTA suppresses BVDV replication both in vitro and in vivo and therefore shows promise as an anti-BVDV agent.

## 1. Introduction

Bovine viral diarrhea virus (BVDV) is a positive-stranded RNA virus of the Pestivirus genus in the family Flaviviridae, which infects cattle, spreads rapidly, and causes major economic losses to the livestock industry worldwide [1,2,3]. BVDV is a prevalent pathogen of cattle that can cause acute disease, immune dysregulation, reproductive failure, respiratory disease, and persistent infections [4]. An economic analysis in 2009 estimated that yearly losses of up to US$88 per animal could be attributed to infection with BVDV [5]. Vaccination has been an essential strategy for the prophylaxis of BVDV infection [6]. Conventional vaccines, namely live-attenuated or inactivated vaccines, have significant shortcomings due to their risk of reversion to virulence or incomplete inactivation [7].

Additionally, they do not allow differentiation of infected from vaccinated animals, leading to economic consequences such as trade restrictions. Effective subunit vaccines which address these challenges are in high demand [8]. However, subunit vaccines based on soluble, monomeric proteins frequently suffer from a limited immunogenicity and require potent adjuvants, high antigen doses, and repetitive vaccinations [9]. 

In recent years, developing effective and safe natural drugs has become a new research direction. Plant polysaccharides have been examined as novel sources of natural compounds for antiviral drug discovery. Bioactive polysaccharides from various medicinal plant sources have different structural features, antiviral capabilities, and mechanisms of action [10]. Forsythiaside A is the main active index component isolated from Forsythiae Fructus, the dried fruit of Forsythia suspensa (Thunb.) Vahl, and possesses prominent bioactivities [11]. The biological activities of FTA are varied, including anti-inflammatory, anti-oxidative, and antiviral functionality properties [11,12]. Moreover, FTA treatment in chicken cells before a viral infection inhibited the replication of avian infectious bronchitis virus [13]. Previous studies in our laboratory have shown that FTA could inhibit the replication of BVDV via TRAF2-dependent CD28-4-1BB signaling in bovine peripheral blood mononuclear cells (PBMCs) [14]. Nonetheless, it is unclear whether FTA could directly inhibit BVDV replication or activate immune responses against BVDV.

The purpose of this paper is to explore the antiviral ability of FTA and the antiviral effect of the combination of FTA and vaccine as an immune enhancer. Herein, by determining the optimal concentration, effective phase against BVDV and potential immunoregulatory effect, we evaluated the anti-BVDV ability of FTA in vitro. We also dissected the immune-enhancing effect of FTA in mice and protective effects of E^rns^-E2 protein vaccine combining with FTA following challenge. Based on the results of these experiments, we propose that FTA suppresses BVDV replication and shows potential for developing novel anti-BVDV drugs.

## 2. Results

### 2.1. FTA Directly Inhibits BVDV Replication in MDBK Cells

**Figure 1:**
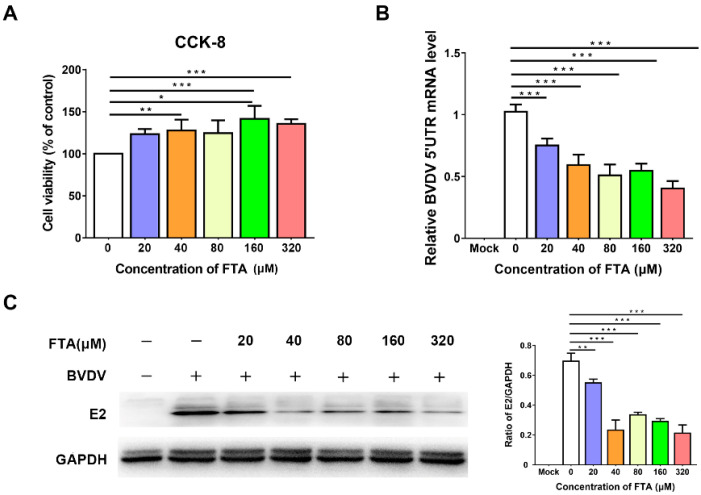

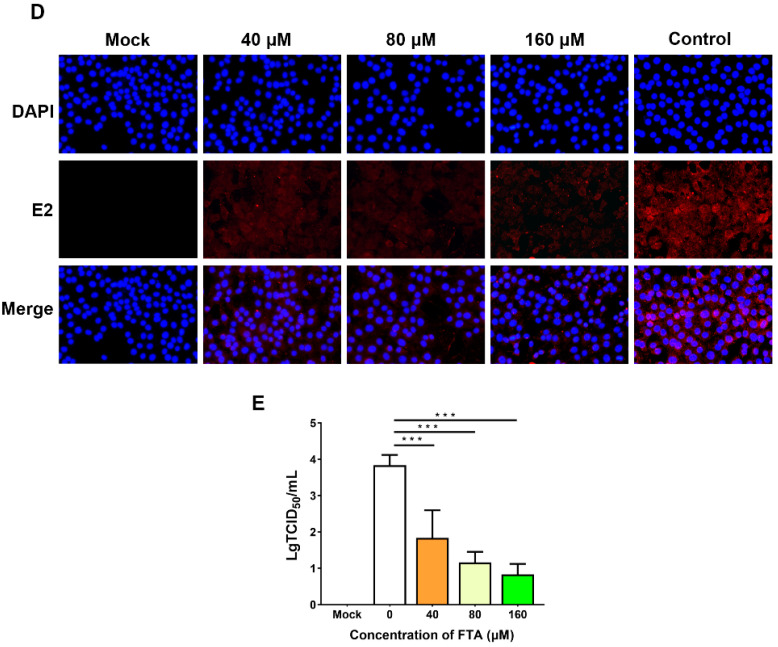
FTA directly inhibits BVDV proliferation in MDBK cells. (**A**) the cytotoxicity of the MDBK cells treated with various concentrations of FTA was determined by a CCK-8 assay. (**B**) 5′UTR mRNA levels in BVDV infected cells treated with FTA were detected by RT-qPCR. (**C**) Lysates of BVDV infected cells treated with FTA were analyzed by immunoblotting. (**D**) IFA of BVDV-infected cells treated with FTA. The cells were fixed and stained with antibodies directed against BVDV E2 protein (red) and DAPI (blue). Scale bar, 50 μm (**E**) The TCID_50_ result for BVDV titers in BVDV infected MDBK cells treated with FTA. Data from three independent experiments and error bars are presented as the mean ± SEM. * *p* < 0.05, ** *p* < 0.01, *** *p* < 0.001.

The effect of FTA on BVDV infectivity was determined in the MDBK cell. First, a CCK-8 assay was performed to determine the cellular cytotoxic effect of FTA in MDBK cells (Figure 1A). The concentration gradient of FTA (20–320 μM) was non-toxic in MDBK cells. Therefore, we next investigated the effect of the concentration of FTA (20–320 μM) on the BVDV infection in MDBK cells. In the presence of FTA, the BVDV E2 protein and mRNA levels were reduced compared with the cells in the BVDV challenge group that were treated with equivalent volumes of DMSO in the Western blotting and RT-qPCR assays (Figure 1B,C). The IFA and TCID_50_ assay indicated that FTA was found to cause a significant decrease in the number of BVDV infected cells and infectious virus particle production (Figure 1D,E). The results show that FTA can directly inhibit BVDV infection in the MDBK cells.

### 2.2. FTA Suppresses BVDV Infection in Different Treatment Modes

**Figure 2:**
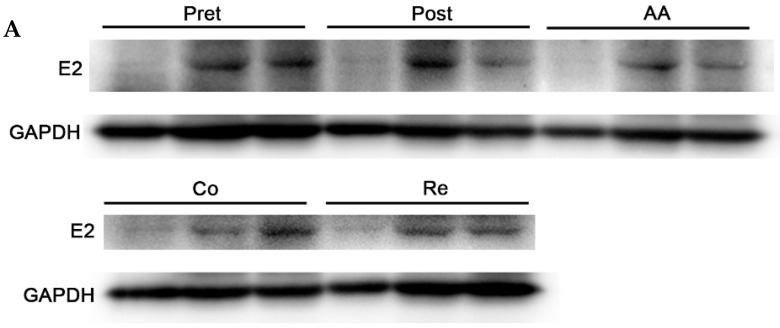

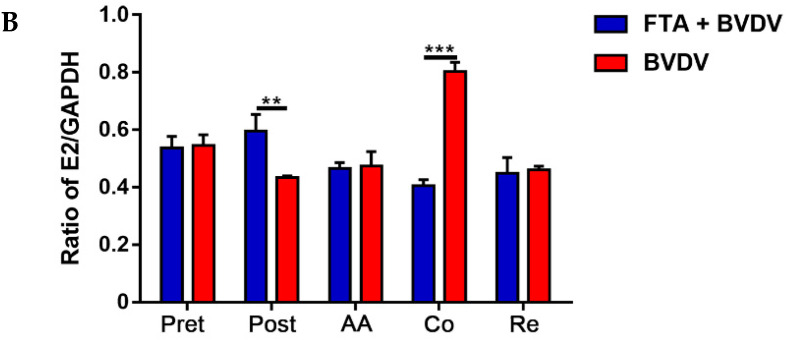
FTA treatment of BVDV infected MDBK cells schemes in the time-of-addition assay. (**A**) Immunoblotting assay was performed to measure the expression of BVDV E2 protein in BVDV-infected MDBK cells treated with FTA under the following conditions: pretreatment, adsorption, posttreatment, cotreatment, and release. (**B**) The protein bands were quantified as the ratio of the intensity of the BVDV E2 protein band to that of the GAPDH band in Western blotting. Data from three independent experiments and error bars are presented as the mean ± SEM. ** *p* < 0.01, *** *p* < 0.001.

To investigate the role of FTA in BVDV life cycle, time-of-addition experiments were performed in MDBK cells under the schemes shown in Figure 2. The Western blotting results showed that the BVDV E2 protein was significantly lower in the FTA cotreatment group than that in the corresponding virus control. The pretreatment, adsorption, posttreatment, and release assay results determined that in the BVDV infected cells treated with FTA, there was no obvious inhibitory effect on the amounts of BVDV E2 protein (Figure 2). Collectively, our data suggested that cotreatment with FTA induces remarkable inhibition on BVDV replication in MDBK cells. 

### 2.3. FTA Attenuates BVDV-Induced Activation of the NLRP3 Inflammasome and Inhibition of the IFN-I in Cells

**Figure 3:**
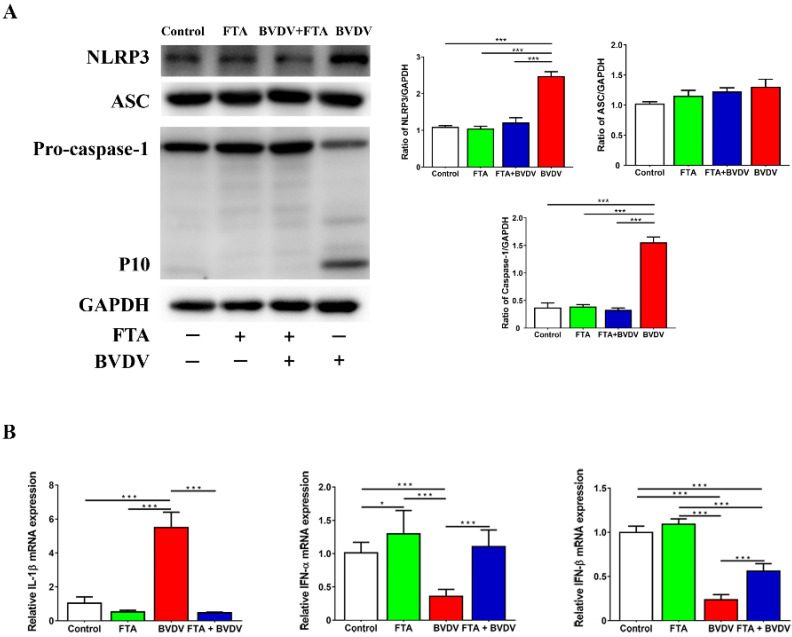
FTA treatment reduced BVDV-induced activation of the NLRP3 inflammasome and increased the IFN-I mRNA. (**A**) Immunoblot analysis of NLRP3, ASC, and Caspase-1 protein expression in cells. (**B**) IFN-α and IFN-β mRNA levels in cells were detected by RT-qPCR. Data from three independent experiments and error bars are presented as the mean ± SEM. * *p* < 0.05, *** *p* < 0.001.

As the most intensively studied inflammasome complex, NLRP3 activated directly interacts with ASC, which then directly interacts with pro-Caspase-1. NLRP3 inflammasome formation activates Caspase-1 and the production of mature IL-1β. In order to explore the effect FTA on the NLRP3 inflammasome in the BVDV infection cells, NLRP3, ASC, Caspase-1 protein, and IL-1β mRNA levels in MDBK cells treated with FTA after 24 h BVDV infection from all groups were performed by Western blotting and RT-qPCR analysis. As shown in the results (Figure 3A,B), the expression of NLRP3, p10 subunit of Caspase-1 protein, and IL-1β mRNA levels in MDBK cells in the BVDV challenge group were significantly higher than that in the mock group. FTA can downregulate the expression of NLRP3, p10 subunit of Caspase-1 protein, and IL-1β mRNA levels in BVDV infected cells than that in the BVDV challenge group (Figure 3A). The expression of ASC protein was not obviously changed in all groups. To determine the effect of FTA on IFN-I in BVDV infection, the IFN-α and IFN-β mRNA levels were detected by RT-qPCR assays in the cells (Figure 3B). The cells in the BVDV challenge group displayed significant reductions in the IFN-α and IFN-β mRNA levels compared with that in the mock group. The BVDV infected cells treated with FTA increased the IFN-α and IFN-β mRNA levels compared with the cells in the BVDV challenge group. The results showed that FTA attenuates BVDV-induced activation of the NLRP3 inflammasome and inhibition of the IFN-I in cells.

### 2.4. T Cell-Mediated Immune Responses and Lymphocyte Proliferation Induced by FTA in Mice

**Figure 4:**
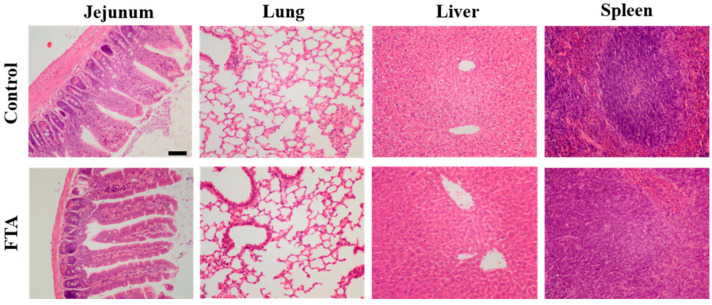
Effects of FTA on the organs of mice. Histopathological changes are observed in the jejunum, lung, liver, and spleen of the mice from each group. Scale bars, 100 μm. Data from three independent experiments and error bars are presented as the mean ± SEM.

**Figure 5:**
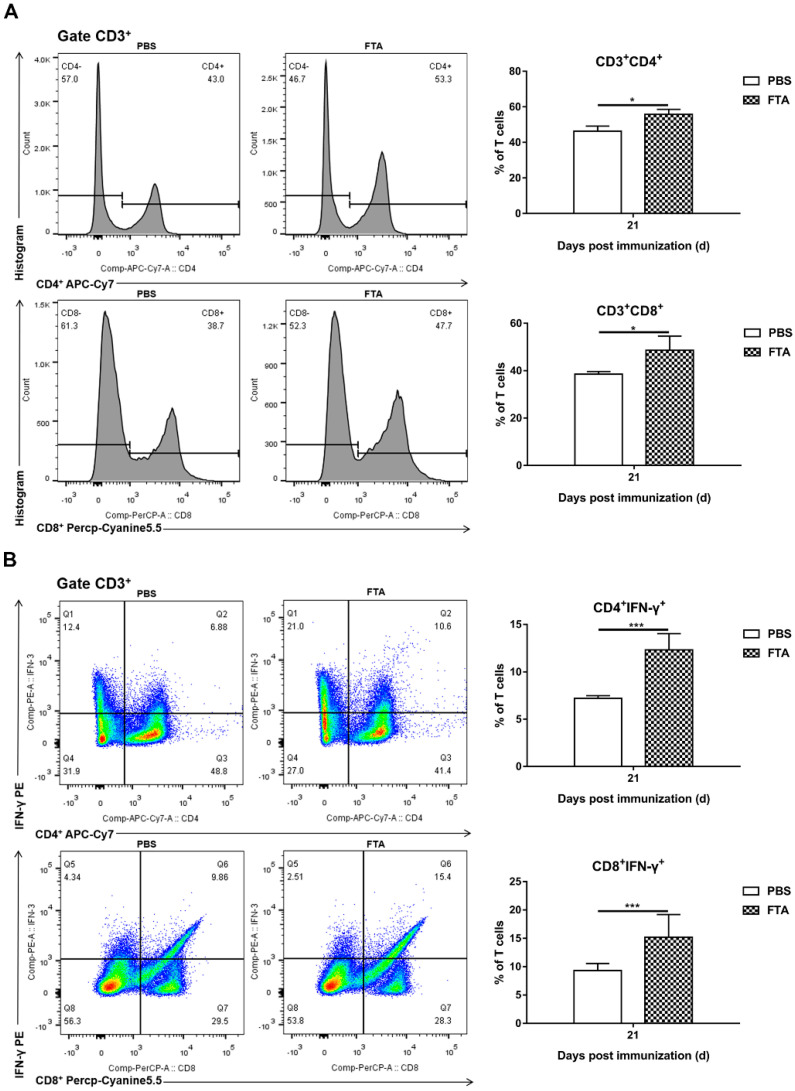
FTA induces the expansion of peripheral blood lymphocytes CD3^+^CD4^+^, CD3^+^CD8^+^, CD4^+^IFN−γ^+^, and CD8^+^IFN−γ^+^ T cells. (**A**) Flow cytometric analysis of the percentage of CD3^+^CD4^+^ and CD3^+^CD8^+^ cells among CD3^+^ T cells. (**B**) Flow cytometric analysis of the percentage of CD4^+^IFN−γ^+^ and CD8^+^IFN−γ^+^cells among CD3^+^ T cells. Left: representative flow cytometry dot plot shows the gating strategy for CD3^+^CD4^+^, CD3^+^CD8^+^, CD4^+^IFN−γ^+^, and CD8^+^IFN−γ^+^ expression in peripheral T cells. Right: The data are the mean percentage of CD3^+^CD4^+^, CD3^+^CD8^+^, CD4^+^IFN−γ^+^, and CD8^+^IFN−γ^+^ cells among CD3^+^ T cells ± SEM of three individual mice from each group detected in triplicate. * *p* < 0.05, *** *p* < 0.001.

**Figure 6:**
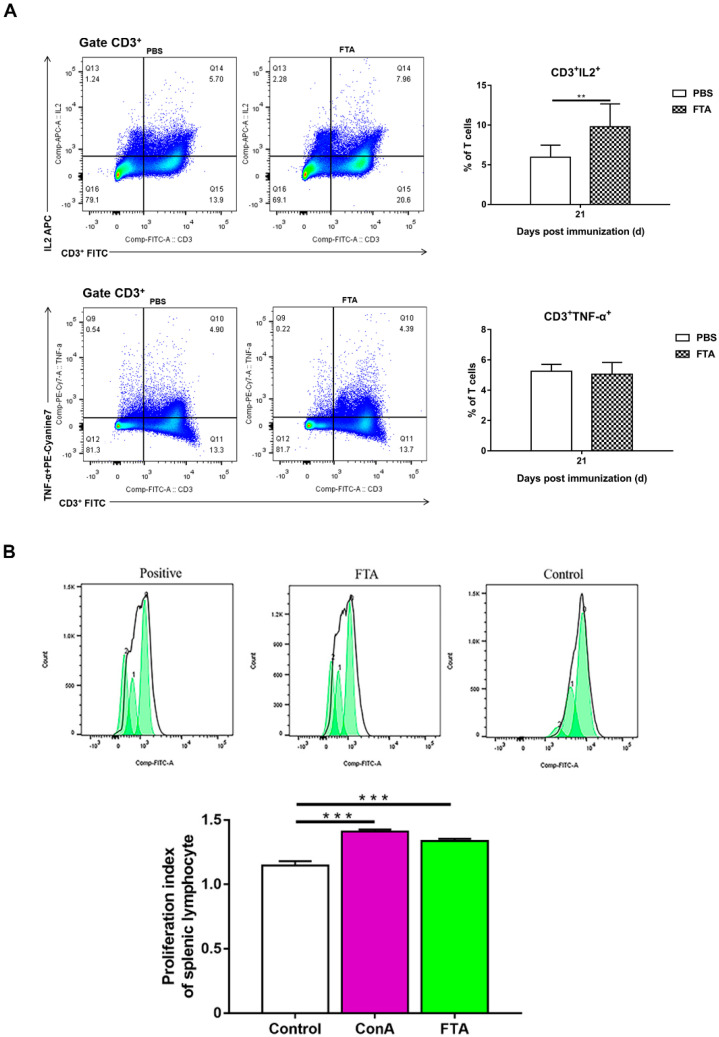
Analysis of peripheral blood lymphocytes CD3^+^IL2^+^ and CD3^+^TNF−α^+^ T cells and splenic lymphocytes proliferation of the mice. (**A**) Flow cytometric analysis of the percentage of CD3^+^IL2^+^ and CD3^+^TNF−α^+^ cells among CD3^+^ T cells. Left: representative flow cytometry dot plot shows the gating strategy for CD3^+^IL2^+^ and CD3^+^TNF−α^+^ expression in peripheral CD3^+^ T cells. Right: The data are the mean percentage of CD3^+^IL2^+^ and CD3^+^TNF−α^+^ T cells among CD3^+^ T cells ± SEM of three individual mice from each group detected in triplicate. ** *p* < 0.01. (**B**) Spleen lymphocyte proliferation of the mice treated with FTA. Left: histogram of splenic lymphocyte proliferation. Right: The data are the mean stimulation index ± SEM of three individual mice from each group detected in triplicate. *** *p* < 0.001.

To evaluate the effects of FTA on various organs in mice, the liver, lung, spleen, and jejunum tissue samples of mice at 21 d treated intraperitoneally with FTA were stained with H&E for morphological examination. We confirmed that no pathological damage was found in the liver, lung, spleen, and jejunum tissue in the FTA treatment mice compared with the control mice (Figure 4). The results demonstrated that the concentration of FTA does not cause damage to the organs of mice in this experiment. In order to assess cellular immunity in mice treated intraperitoneally with FTA, we analyzed the proportion of cells named CD3^+^CD4^+^, CD3^+^CD8^+^, CD3^+^CD4^+^IFN-γ^+^, CD3^+^CD8^+^IFN-γ^+^, CD3^+^IL-2^+^, and CD3^+^TNF-α^+^ in peripheral blood lymphocytes of mice at 21 d by flow cytometry. As shown in Figure 5A,B and Figure 6A, the FTA treatment group reported significantly higher counts of CD3^+^CD4^+^, CD3^+^CD8^+^, CD3^+^CD4^+^IFN-γ^+^, CD3^+^CD8^+^IFN-γ^+^, and CD3^+^IL-2^+^ lymphocytes than the control group, whereas CD3^+^TNF-α^+^T cells in the mice treated with FTA were not differenced with the control group. Furthermore, the results of the spleen lymphocyte proliferation are shown in Figure 6B. The proliferation index of splenic lymphocytes in the FTA group was markedly higher than in the control group.

### 2.5. FTA Contributes to Reducing Tissues Damage in BVDV-Infected Mice

**Figure 7:**
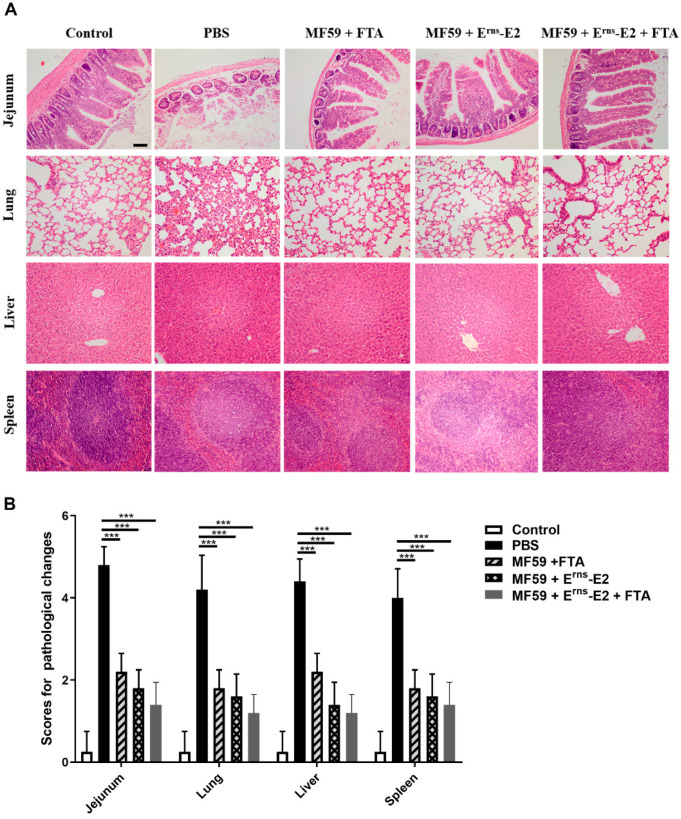
The histopathological changes and scores of pathological changes after challenge following the recombinant E^rns^-E2 protein vaccine-FTA adjuvant combination treatment. (**A**) Histopathological changes were observed in the lungs, spleen, and intestines of the mice from each group. Scale bars, 100 μm. (**B**) Scores of pathological changes in tissues of mice. Pathological changes in tissues were evaluated by a veterinary pathologist and scored from 0 to 5 in a blinded study. Data from three independent experiments and error bars are presented as the mean ± SEM. *** *p* < 0.001.

To evaluate whether FTA as an immune enhancer can synergize with vaccines to reduce virus damage to the tissues of mice at 35 d, the lung, liver, spleen, and intestine collected from mice subjected to HE staining (Figure 7). The histopathology results showed that the lung, liver, spleen, and intestine displayed the most severe pathological changes among the PBS group after infection; the intestinal tissue changes in the PBS group were characterized by intestinal villi tip breakage, erosion, shedding of intestinal epithelial cells, flattened crypt epithelium, and loosening and edema of the submucosa; the lung tissue changes in the PBS group exhibited alveolar expansion, alveolar wall thickening, and lymphocyte infiltration; the liver tissue changes in the PBS group showed damaged structures of the liver tissue, a disordered arrangement of the liver cells, and hepatocyte degeneration and necrosis; the spleen tissue changes in the PBS group displayed the damaged spleen structure, lymphocyte decrease in the lymphatic nodule and macrophage increase compared with that in the other groups (Figure 7). 

The tissues in the MF59 + FTA group demonstrated mild pathological changes compared with that of the negative control group; the lung tissue changes were characterized by blood vessel congestion or mild alveolar expansion. The jejunums, liver, and spleen tissue in the MF59 + FTA group had no obvious pathological changes (Figure 7).

The tissues in the negative control group, the MF59 + E^rns^-E2 group, and the MF59 + E^rns^-E2 + FTA group had no obvious pathological changes (Figure 7). Scores for pathological lesions in BVDV-infected mice in each group are shown in Figure 7B. Taken together, these results suggest the recombinant E^rns^-E2 protein vaccine-FTA adjuvant combination could clearly reduce tissue damage in BVDV-infected mice.

### 2.6. BVDV-Specific Antibody Responses Induced by FTA in Mice

**Figure 8:**
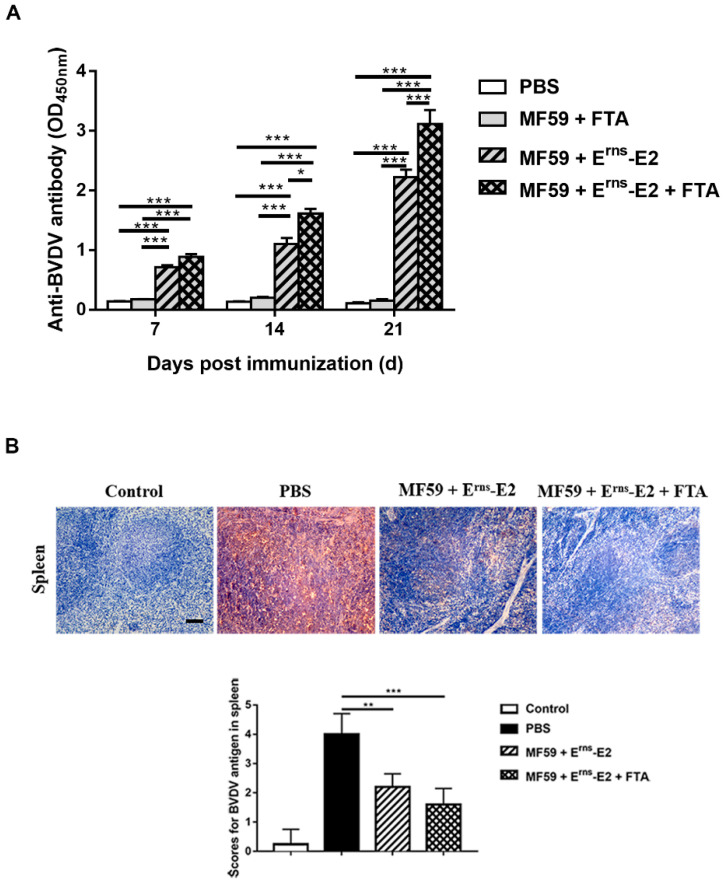
BVDV-specific antibody responses and the viral load of spleen tissue of mice after BVDV challenge following the recombinant E^rns^-E2 protein vaccine-FTA adjuvant combination treatment. (**A**) The neutralizing antibodies were detected in different experimental groups. Each sample was independently tested thrice. (**B**) The distribution of BVDV antigen in the spleens of mice was determined by IHC. Scale bar, 100 μm. Data from three independent experiments and error bars are presented as the mean ± SEM. * *p* < 0.05, ** *p* < 0.01, *** *p* < 0.001.

The levels of specific antibodies were measured in the sera to evaluate the effects of FTA on humoral immunization responses. In the E^rns^-E2 protein formulated with FTA immunized group, mice induced at 7, 14, and 21 d after primary immunization had significantly higher levels of BVDV IgG responses compared with mice of the other groups (Figure 8A). Notably, antibody titers trended upward as the immunization process continued. The results showed that FTA could significantly increase the production of antibody titers in immunized mice.

### 2.7. FTA Reduces the Viral Load of Spleen Tissues of Mice Infected with BVDV

The splenic lymphocytes of the mice were assessed by immunohistochemistry (IHC) to evaluate the effects of FTA on the viral antigen burden in the mice in each group at 35 d after the BVDV challenge (Figure 8B). BVDV antigens were observed throughout the lymphocyte in the spleen of mice from the PBS group after BVDV infection, whereas in the negative control group, no positive signals were detected by IHC. BVDV antigen-positive cells in the MF59 + E^rns^-E2 group and MF59 + E^rns^-E2 + FTA group decreased compared with that of the PBS group after infection, and positive signals were least in the MF59 + E^rns^-E2 + FTA group (Figure 8B). The scores of BVDV antigens showed a significant difference in the spleens of mice in each group (Figure 8B). In summary, our data suggest that the recombinant E^rns^-E2 protein vaccine-FTA adjuvant combination could markedly inhibit the BVDV infection in mice.

## 3. Discussion

BVDV spreads widely worldwide, causing substantial annual losses to the dairy farming industry [2,15]. At present, there are no safe and effective commercial vaccines or drugs against BVDV. Considering that FTA could protect PBMCs from BVDV infection via TRAF2-dependent CD28-4-1BB signaling [14], we aimed to explore the possible mechanism of inhibition of BVDV replication by FTA and evaluate the efficacy of the recombinant E^rns^-E2 protein vaccine-FTA adjuvant combination. In this study, our results demonstrate that FTA can play an immune-enhancing effect in the form of adjuvant in vivo to improve the immune effect of the vaccine, significantly inhibit virus replication, and delay the occurrence of virus infection in model animals.

In general, the inhibitory effect of natural plant extracts on viruses may be reflected in the inhibition of virus adsorption to host cells [16]. For example, Taishan Pinus massoniana pollen polysaccharide can interfere with avian leukosis virus subgroup J adsorption, and the possible mechanism is the direct interaction between TPPPS and the viral envelope protein gp85 [17]. Baicalein prevents the entry of human cytomegalovirus into cells by affecting the activity of targeted epidermal growth factor receptor kinases [18,19]. However, our results suggest that FTA seems to promote the invasion of BVDV particles into MDBK cells (Figure 2), which may be related to the fact that FTA may increase the number of LDLR, CD46, and claudin-related receptors by promoting the proliferation of MDBK cells. It has been reported that LDLR, CD46, and claudin-related receptors are crucial to internalization in BVDV infection [20,21]. The pretreatment results showed that FTA could not directly destroy or kill BVDV.

Various factors such as viruses, bacteria, and fungi can activate NLRP3 and cause pyroptosis [22]. IFN-I is an important cytokine that exerts antiviral effects. Double-stranded RNA, an intermediate product produced during virus replication in cells, is a strong inducer of IFN-I production [23]. NCP-BVDV can inhibit the production of IFN-I induced by double-stranded RNA after infection [24,25]. Our findings found that FTA could not only inhibit BVDV infection-induced pyroptosis by reducing the functional activation of NLRP3, but also upregulate the expression of type I IFN, which is consistent with the fact that FTA can significantly inhibit viral replication (Figure 3). The results indicate that FTA can exert antiviral effects by inhibiting BVDV-induced pyroptosis and promoting the expression of IFN-I. These data may explain why the antiviral effect of FTA required treatment at several stages of BVDV infection rather than a single stage.

Based on the fact that FTA can interfere with BVDV infection in MDBK cells in vitro (Figure 1, Figure 2 and Figure 3), we further verified the immune-enhancing effect of FTA and the antiviral effect of synergistic immunization with vaccines in vivo (Figure 4, Figure 5, Figure 6, Figure 7 and Figure 8). Immune cells can defend against microbial pathogens and infectious diseases. Changes in the proportion of lymphocytes can reflect the overall immune status of the body, as well as cellular and humoral immunity [26,27,28]. CD4^+^ and CD8^+^ are two important surface markers of T cells. CD4^+^ T cells can induce and enhance the body’s immune response and stimulate the activation and proliferation of B cells by secreting various immunologically active cytokines. Specific neutralizing antibodies play an essential role in the body’s immune system [29,30]. CD8^+^ T cells are a principal component of immunity in resisting and clearing intracellular pathogens such as viruses [27,31]. In the study, the percentages of CD3^+^CD4^+^ and CD3^+^CD8^+^ T cells in the peripheral blood of the FTA group were significantly higher than those of the control group, indicating that FTA can stimulate the proliferation and activation of T cells and enhance cellular immunity (Figure 5A). In addition, IFN-γ is secreted by Th1-type cells, which mainly promote cellular immune responses and play a vital role in regulating the immune system and broad-spectrum antiviral functions [32]. The results showed that the percentages of CD4^+^ IFN-γ^+^ and CD8^+^ IFN-γ^+^ T lymphocytes in the peripheral blood of the FTA group were significantly higher than those of the control group, indicating that FTA can enhance the Th1-type immune response (Figure 5B). IL-2 is a B-cell growth factor, stimulates antibody synthesis, and promotes lymphocyte growth, proliferation, and differentiation [33,34]. We found that FTA increased the percentage of CD3^+^IL-2^+^ T lymphocytes in peripheral blood of mice and promoted the proliferation of spleen lymphocytes (Figure 6). After immunization, by combining FTA with MF59 + E^rns^-E2 protein vaccine, FTA can stimulate higher levels of specific antibodies in the body (Figure 8A). Our findings suggest that FTA can promote the secretion of IL-2, that act as a B cell growth factor to stimulate the synthesis of antibodies [34]. This may be the reason why FTA plays a role in producing higher levels of BVDV-specific antibodies.

The results found that the mice in the MF59 + E^rns^-E2 + FTA immunized group had the least pathological damage and viral load in the spleen tissue after the challenge compared with other groups (Figure 7 and Figure 8B). This may be related to the fact that FTA promotes the activation of CD4^+^ T lymphocytes, thereby promoting Th1-type cells to secrete IFN-γ and IL-2 cytokines and stimulate the production of high levels of specific antibodies in the body (Figure 5 and Figure 6). In addition, FTA can promote the proliferation and activation of CD8^+^IFN-γ^+^ T lymphocytes and benefit the body to clear and resist BVDV.

Taken together, this study determined the optimal concentration, mode of action, and mechanism of action of FTA to inhibit BVDV proliferation in MDBK cells. Strikingly, FTA can reduce the activation of NLRP3 to inhibit the inflammatory response caused by BVDV infection and upregulate the expression of IFN-I to clear the virus. FTA can stimulate the proliferation and activation of T lymphocytes in peripheral blood of mice and enhance cellular immunity. At the same time, our finding also sheds light on how FTA can improve the antiviral effect of the vaccine when it is synergistically immunized with the vaccine.

## 4. Materials and Methods

### 4.1. Virus, Cells, and Reagents

Madin–Darby Bovine Kidney (MDBK) cells were cultured in Dulbecco’s Modified Eagle Medium/Ham’s F-12 medium (1:1) (Gibco, Grand Island, NY, USA) supplemented with 7% fetal bovine serum (Thermo Scientific, Waltham, MA, USA) at 37 °C in a humidified atmosphere containing 5% CO_2_.

The NCP-BVDV-BJ175170 isolate strain, which was isolated from blood samples from cows with suspected BVDV infections, was maintained in our laboratory. The BVDV BJ175170 isolate strains were utilized for all experiments and are represented by “BVDV” in this article.

FTA (purity > 99%) was purchased from Selleck (Houston, TX, USA), solubilized in 100% DMSO, and kept frozen as 10 mM stocks. The compounds were stored in small aliquots to prevent multiple freeze-thaws and were stepwise diluted to reach the desired concentration in 0.5% DMSO for all treatments. Anti-NLRP3 antibody and anti-ASC antibody, anti-Caspase-1 antibody, anti-Tubulin antibody (ProteinTech Group, Rosemont, IL, USA), and anti-BVDV E2-protein mouse monoclonal antibody (mAb) (VMRD, Pullman, WA, USA) were used in the study.

### 4.2. Drug Treatment

MDBK cells were plated in a 6-well culture plate. The cells were treated with 0.5% DMSO or the FTA (final concentrations were 20, 40, 80, 160, and 320 μM) and infected with BVDV (MOI = 0.1) for 2 h. The virus inoculum was removed 2 h after infection and replaced with complete medium with the FTA indicated a range of concentrations or 0.5% DMSO. The mock group was untreated. The antiviral activity of the FTA was assessed by detecting the replication of the virus in the infected cells.

### 4.3. Cell Viability Assay 

MDBK cells were treated with complete DMEM supplemented with 0.5% DMSO with FTA (20, 40, 80, 160, and 320 μM) or 0.5% DMSO alone.for 48 h at 37 °C. Cell viability was tested with a Cell Counting Kit-8 (CCK-8) (Beyotime Biotechnology, Shanghai, China) according to the manufacturer’s instructions. 

### 4.4. Indirect Immunofluorescence Assay (IFA) 

The effect of FTA on MDBK cells infected with BVDV was detected with an IFA. The cells were incubated with 4% paraformaldehyde for 15 min and then incubated in 0.1% Triton X-100 for permeabilization. Anti-BVDV E2-protein (1:1000) mAb diluted in BSA (1%) were added to the cells for 1.5 h at 37 °C. Alexa Fluor 555-conjugated goat anti-rabbit IgG (Zhongshan Golden Bridge Biotechnology, Beijing, China) was applied as the second antibody for visualization of the virus existence. The nuclei were stained with 4′, 6-diamidino-2-phenylindole (DAPI, 100 ng/mL) for 5 min. Immunofluorescence was observed under a Nikon Eclipse Ti-U in-verted fluorescence microscope (Nikon, Tokyo, Japan).

### 4.5. Western Blotting 

Proteins were extracted from MDBK cells for Western blot assay. Anti-BVDV E2-protein (1:1000), anti-GAPDH (1:5000), anti-Tubulin (1:5000), anti-NLRP3 (1:1000), anti-ASC polyclonal (1:1000), and rabbit anti-Caspase-1 (1:1000) were used as the primary antibodies. Horseradish-peroxidase-conjugated goat anti-mouse and anti-rabbit IgG antibodies (1:5000; Proteintech, Rosemont, IL, USA) were used as the secondary antibodies. The immunoreactivity was detected using Chemistar High-sig ECL Western blotting substrate (Tanon, Shanghai, China) and visualized on a Tanon 5200 system (Tanon, Shanghai, China).

### 4.6. RNA Extraction and Quantitative Reverse Transcription PCR (RT-qPCR)

The total RNA was extracted from MDBK cells using the RNAiso Plus (Takara, Kyoto, Japan) and then reverse transcribed into cDNA using a PrimeScript™ RT reagent Kit with a gDNA Eraser (Takara, Kyoto, Japan) according to the manufacturer’s instructions. The procedures for analysis of gene expression were described previously [35]. The 5′UTR, interferon (IFN)-α, IFN-β, interleukin (IL)-1β and GAPDH genes specific primers used were listed in Table 1. Expression of the genes was measured by the relative quantification method. Sampling was performed in triplicate in each reaction, and the data were calculated as the fold-change using the 2^−ΔΔCT^ method.

### 4.7. Viral Titration

The effect of FTA on BVDV viral titration in MDBK cells was determined by TCID_50_. The cells were incubated with 10-fold serial dilutions of the supernatants of BVDV infection cells treated with FTA or 0.5% DMSO in eight replicates in the 96-well plates. The plates were assessed with IFA. Immunofluorescence was observed using a Nikon Eclipse Ti-U inverted fluorescence microscope. BVDV titers were calculated using the Reed–Muench method.

### 4.8. Time-of-Addition Assay

The- time- of addition assay was performed to detect the effect of FTA (160 μM) treatment on BVDV (MOI = 0.1) infection. Pretreatment assay: BVDV was mixed with FTA and incubated for 1 h. The cells were incubated in the mixture for 1 h. Adsorption assay: The cells were simultaneously incubated with FTA and BVDV. One hour later, the supernatant was replaced with a compound-free medium. Posttreatment assay: The cells were first infected with BVDV for 1 h then incubated in fresh medium containing FTA. Cotreatment assay: The cells were incubated with FTA for 1 h and then infected with BVDV for 1 h. The virus–FTA mixture was removed and incubated with a fresh medium including FTA. Release assay: After the cells were infected with BVDV for 2 h, the cells were incubated with FTA. The DMSO group was the infection control group. The cells were harvested at 24 h for a Western blotting analysis.

### 4.9. Animal Design

One hundred, 6–8 weeks-old SPF female BALB/c mice were purchased from Beijing Vital River Laboratory Animal Technology Co., Ltd. (Beijing, China) and maintained in negative-pressure isolators. Two experiment plans were designed to assess both immune stimulation (Exp-1, group I to II) and the preventive potentials (Exp-2, group III to VI) of FTA. In Exp-1, twenty of the mice were randomly divided into two groups. The mice in group I and II were treated intraperitoneally with PBS and FTA at 0, 7, and 14 days (d).

The mice in group III to VI were randomly divided into four groups (twenty animals of each group) and immunized three times intraperitoneally as follows: group III, PBS; group IV, MF59 adjuvant and FTA (60 mg/kg FTA); group V, 60 μg of recombinant E^rns^-E2 protein containing MF59 adjuvant vaccine; group VI, 60 μg of recombinant E^rns^-E2 protein containing MF59 adjuvant and FTA vaccine. All mice in groups III to VI were infected with the BVDV at 28 the first day post-hatching (dph). The blood and tissues were collected on the 7, 14, and 21 d and on the 35 d, respectively, after vaccine antibody detection in the mice. All mice were humanely euthanized at the end of the experiment. The process of vaccination and sample collection are shown in Figure S1.

### 4.10. Flow Cytometry

Peripheral blood mononuclear cells (PBMCs) isolated from the blood plasma of mice at 21 d were purified and collected as previously described [9]. Cells were stained with following monoclonal antibodies: fluorescein isothiocyanate–conjugated anti-mouse CD3e (clone 145-2C11, 11-0311-82, Thermo Fisher Scientific, Waltham, MA, USA), Percp-Cyanine5.5-conjugated anti-mouse CD8 (clone 53- 6.7, 5-0081-80, Thermo Fisher Scientific, Waltham, MA, USA), Apc-Cy7-conjugated anti-mouse CD4 (clone GK15, 561830, BD Biosciences, San Jose, CA, USA), APC-conjugated anti-mouse IL-2 (clone JES6-5H4, 17-7021-81, Thermo Fisher Scientific, Waltham, MA, USA), and PE-Cyanine7-conjugated anti-mouse TNF-α (clone MP6-XT22, 25-7321-82, Thermo Fisher Scientific) in the dark for 30 min at 4 °C. After washing with staining buffer, fixing in Cytofix/Cytoperm and permeabilizing with Perm/Wash buffer (BD Biosciences, San Jose, CA, USA), cells were then intracellularly stained with anti-mouse IFN-γ (clone XMG1.2, PE-conjugated, 12-7311-82, Thermo Fisher Scientific, Waltham, MA, USA) in the dark for 30 min at 4 °C. The antibody-stained cells were determined by a FACScalibur™ flow cytometer (BD Biosciences, San Jose, CA, USA). FlowJo 9.3 software (Tree Star, Ashland, OR, USA) was used to perform the data analysis.

### 4.11. Spleen Lymphocyte Proliferation

Cells were isolated from the spleen of each mouse in each group on aseptic conditions at 21 d after the FTA treatment. Erythrocyte lysate type B 1X (product # 181106X, Tiandz, Beijing, China) was added to the mouse spleen cells to lyse erythrocytes. The splenic leucocytes labeled with BD Horizon™ CFSE (1 μM) for 10 min at 37 °C and then resuspended in complete RPMI 1640 medium. The cells were incubated with the T/B-cell specific mitogen ConA (20 μg/mL) (Sigma-Aldrich, St. Louis, MO, USA), FTA or PBS respectively. The cells were analyzed by flow cytometry after three days.

### 4.12. Histology and Immunohistochemistry

The liver, lung, spleen, and jejunum tissue samples of euthanized mice at 35 d taken from group were collected and immersed in 4% formalin for 48 h. The collected samples were successively trimmed, embedded in paraffin, and cut into 4 μm sections. Sections of pathological tissue were stained with hematoxylin and eosin (H&E). Meanwhile, for examining to BVDV viral antigen in spleen tissue samples, sections of pathological tissue were performed with immunohistochemistry. The experimental program and scored were as previously described [9].

### 4.13. Elisa (Enzyme Linked Immunosorbent Assay)

To assess the titers of serum anti-BVDV antibody in mice, the serum samples were selected from each group at 7, 14, and 21d.The anti-BVDV Immunoglobulin G (IgG) antibody titers in the sera of immunized mice were determined in accordance with the previous reference [9].

### 4.14. Statistical Analysis

Data are shown as mean ± standard deviation and were analyzed using SPSS (Statistical Package for the Social Sciences, version 17.0, Chicago, IL, USA). The statistical significance of differences amongst the mean values of multiple groups were evaluated with a one-way analysis of variance (ANOVA) or a two-tailed Student *t*-test. *p*-value < 0.05, *p*-value < 0.01, and *p*-value < 0.001 were considered statistically significant.

## Figures and Tables

**Table 1:** Sequences of primers used for RT-qPCR.

Primer Name	Primer Sequence(5′–3′)
5′UTR F	TAGTCGTCAGTGGTTCACGCC
5′UTR R	CCTCTGCAGCACCCTATCAG
IL-1β F	AGTGCCTACGCACATGTCTTC
IL-1β R	TGCGTCACACAGAAACTCGTC
IFN-α F	GTGAGGAAATACTTCCACAGACTCACT
IFN-α R	GAGGAAGAGAAGGCTCTCATGA
IFN-β F	CCTGTGCCTGATTTCATCATGA
IFN-β R	GCAAGCTGTAGCTCCTGGAAAG
GAPDH F	AAAGTGGACATCGTCGCCAT
GAPDH R	CCGTTCTCTGCCTTGACTGT

## Data Availability

Not applicable.

## Supplementary Materials

The following supporting information can be downloaded at: https://www.mdpi.com/article/10.3390/ijms23169390/s1.

## Abbreviations

ASC: Apoptosis-associated speck-like protein containing a CARD; BVDV: Bovine viral diarrhea virus; CCK-8: Cell Counting Kit-8; CFSE: carboxyfluorescein diacetate succinimidyl ester; ConA: Concanavalin A; DAPI; 4′, 6-diamidino-2-phenylindole; DMSO: dimethyl sulfoxide; Elisa: Enzyme linked immunosorbent assay; Exp-1: experiment-1; FBS: Fetal Bovine Serum; FTA: Forsythoside A; GAPDH: glyceraldehyde-3-phosphate dehydrogenase; HE: hematoxylin and eosin; IFA: Indirect immunofluorescence assay; IFN-I: type I interferon; IL-1β: interleukin-1β; LDLR: Low-density lipoprotein receptor; mAB: monoclonal antibody; MDBK: Madin–Darby Bovine Kidney; MOI: multiplicity of infection; NCP: non-cytopathic; NLRP3: Nucleotide binding and Leucine rich repeat Receptors 3; PBMCs: peripheral blood mononuclear cells; PBS: phosphatebuffered saline; RT-qPCR: quantitative reverse transcription PCR; TCID_50_: tissue culture infectious dose 50; TRAF2: TNF receptor-associated factor 2; UTR: untranslated regions.

## References

[1] Richter V., Lebl K., Baumgartner W., Obritzhauser W., Kasbohrer A., Pinior B. (2017). A systematic worldwide review of the direct monetary losses in cattle due to bovine viral diarrhoea virus infection. Vet. J..

[2] Yesilbag K., Alpay G., Becher P. (2017). Variability and Global Distribution of Subgenotypes of Bovine Viral Diarrhea Virus. Viruses.

[3] Bollini M., Leal E.S., Adler N.S., Aucar M.G., Fernandez G.A., Pascual M.J., Merwaiss F., Alvarez D.E., Cavasotto C.N. (2018). Discovery of Novel Bovine Viral Diarrhea Inhibitors Using Structure-Based Virtual Screening on the Envelope Protein E2. Front. Chem..

[4] Pinior B., Garcia S., Minviel J.J., Raboisson D. (2019). Epidemiological factors and mitigation measures influencing production losses in cattle due to bovine viral diarrhoea virus infection: A meta-analysis. Transbound. Emerg. Dis..

[5] Pinior B., Firth C.L. (2017). The economics of bovine viral diarrhoea eradication. Vet. Rec..

[6] Richter V., Kattwinkel E., Firth C.L., Marschik T., Dangelmaier M., Trauffler M., Obritzhauser W., Baumgartner W., Kasbohrer A., Pinior B. (2019). Mapping the global prevalence of bovine viral diarrhoea virus infection and its associated mitigation programmes. Vet. Rec..

[7] Al-Kubati A., Hussen J., Kandeel M., Al-Mubarak A., Hemida M.G. (2021). Recent Advances on the Bovine Viral Diarrhea Virus Molecular Pathogenesis, Immune Response, and Vaccines Development. Front. Vet. Sci..

[8] Falkenberg S.M., Dassanayake R.P., Terhaar B., Ridpath J.F., Neill J.D., Roth J.A. (2021). Evaluation of Antigenic Comparisons Among BVDV Isolates as it Relates to Humoral and Cell Mediated Responses. Front. Vet. Sci..

[9] Wang S., Yang G., Nie J., Yang R., Du M., Su J., Wang J., Wang J., Zhu Y. (2020). Recombinant E(rns)-E2 protein vaccine formulated with MF59 and CPG-ODN promotes T cell immunity against bovine viral diarrhea virus infection. Vaccine.

[10] Xie J.H., Jin M.L., Morris G.A., Zha X.Q., Chen H.Q., Yi Y., Li J.E., Wang Z.J., Gao J., Nie S.P. (2016). Advances on Bioactive Polysaccharides from Medicinal Plants. Crit. Rev. Food Sci. Nutr..

[11] Gong L., Wang C., Zhou H., Ma C., Zhang Y., Peng C., Li Y. (2021). A review of pharmacological and pharmacokinetic properties of Forsythiaside A. Pharmacol. Res..

[12] Law A.H., Yang C.L., Lau A.S., Chan G.C. (2017). Antiviral effect of forsythoside A from Forsythia suspensa (Thunb.) Vahl fruit against influenza A virus through reduction of viral M1 protein. J. Ethnopharmacol..

[13] Wang X., Li X., Wang X., Chen L., Ning E., Fan Y., Wang H., Chen T., Wang W. (2021). Experimental study of Forsythoside A on prevention and treatment of avian infectious bronchitis. Res. Vet. Sci..

[14] Song Q.J., Weng X.G., Cai D.J., Zhang W., Wang J.F. (2016). Forsythoside A Inhibits BVDV Replication via TRAF2-Dependent CD28-4-1BB Signaling in Bovine PBMCs. PLoS ONE.

[15] Deng M., Chen N., Guidarini C., Xu Z., Zhang J., Cai L., Yuan S., Sun Y., Metcalfe L. (2020). Prevalence and genetic diversity of bovine viral diarrhea virus in dairy herds of China. Vet. Microbiol..

[16] Wolkerstorfer A., Kurz H., Bachhofner N., Szolar O.H. (2009). Glycyrrhizin inhibits influenza A virus uptake into the cell. Antivir. Res..

[17] Yu C., Wei K., Liu L., Yang S., Hu L., Zhao P., Meng X., Shao M., Wang C., Zhu L. (2017). Taishan Pinus massoniana pollen polysaccharide inhibits subgroup J avian leucosis virus infection by directly blocking virus infection and improving immunity. Sci. Rep..

[18] Cotin S., Calliste C.A., Mazeron M.C., Hantz S., Duroux J.L., Rawlinson W.D., Ploy M.C., Alain S. (2012). Eight flavonoids and their potential as inhibitors of human cytomegalovirus replication. Antivir. Res..

[19] Evers D.L., Chao C.F., Wang X., Zhang Z., Huong S.M., Huang E.S. (2005). Human cytomegalovirus-inhibitory flavonoids: Studies on antiviral activity and mechanism of action. Antivir. Res..

[20] Riedel C., Chen H.W., Reichart U., Lamp B., Laketa V., Rumenapf T. (2020). Real Time Analysis of Bovine Viral Diarrhea Virus (BVDV) Infection and Its Dependence on Bovine CD46. Viruses.

[21] Krey T., Moussay E., Thiel H.J., Rumenapf T. (2006). Role of the low-density lipoprotein receptor in entry of bovine viral diarrhea virus. J. Virol..

[22] He Y., Hara H., Nunez G. (2016). Mechanism and Regulation of NLRP3 Inflammasome Activation. Trends Biochem. Sci..

[23] Wu S.F., Xia L., Shi X.D., Dai Y.J., Zhang W.N., Zhao J.M., Zhang W., Weng X.Q., Lu J., Le H.Y. (2020). RIG-I regulates myeloid differentiation by promoting TRIM25-mediated ISGylation. Proc. Natl. Acad. Sci. USA.

[24] Schweizer M., Matzener P., Pfaffen G., Stalder H., Peterhans E. (2006). “Self” and “nonself” manipulation of interferon defense during persistent infection: Bovine viral diarrhea virus resists alpha/beta interferon without blocking antiviral activity against unrelated viruses replicating in its host cells. J. Virol..

[25] Schweizer M., Peterhans E. (2001). Noncytopathic bovine viral diarrhea virus inhibits double-stranded RNA-induced apoptosis and interferon synthesis. J. Virol..

[26] Jones D.M., Read K.A., Oestreich K.J. (2020). Dynamic Roles for IL-2-STAT5 Signaling in Effector and Regulatory CD4^+^ T Cell Populations. J. Immunol..

[27] Laidlaw B.J., Craft J.E., Kaech S.M. (2016). The multifaceted role of CD4^+^ T cells in CD8^+^ T cell memory. Nat. Rev. Immunol..

[28] Zhu J., Yamane H., Paul W.E. (2010). Differentiation of effector CD4 T cell populations. Annu. Rev. Immunol..

[29] Hong S., Zhang Z., Liu H., Tian M., Zhu X., Zhang Z., Wang W., Zhou X., Zhang F., Ge Q. (2018). B Cells Are the Dominant Antigen-Presenting Cells that Activate Naive CD4^+^ T Cells upon Immunization with a Virus-Derived Nanoparticle Antigen. Immunity.

[30] Bedoui S., Heath W.R., Mueller S.N. (2016). CD4^+^ T-cell help amplifies innate signals for primary CD8^+^ T-cell immunity. Immunol. Rev..

[31] Janssen E.M., Lemmens E.E., Wolfe T., Christen U., von Herrath M.G., Schoenberger S.P. (2003). CD4^+^ T cells are required for secondary expansion and memory in CD8^+^ T lymphocytes. Nature.

[32] Akdis M., Aab A., Altunbulakli C., Azkur K., Costa R.A., Crameri R., Duan S., Eiwegger T., Eljaszewicz A., Ferstl R. (2016). Interleukins (from IL-1 to IL-38), interferons, transforming growth factor beta, and TNF-alpha: Receptors, functions, and roles in diseases. J. Allergy Clin. Immunol..

[33] Spolski R., Li P., Leonard W.J. (2018). Biology and regulation of IL-2: From molecular mechanisms to human therapy. Nat. Rev. Immunol..

[34] Boyman O., Sprent J. (2012). The role of interleukin-2 during homeostasis and activation of the immune system. Nat. Rev. Immunol..

[35] Yang G., Zhang J., Wang S., Wang J., Wang J., Zhu Y., Wang J. (2021). Gypenoside Inhibits Bovine Viral Diarrhea Virus Replication by Interfering with Viral Attachment and Internalization and Activating Apoptosis of Infected Cells. Viruses.

