# Supplementary material for: Forsythiaside A Improves the Inhibitory Efficiency of Recombinant Protein Vaccines against Bovine Viral Diarrhea Virus Infection
Source: Int J Mol Sci. 2022 Aug 20;23(16):9390. doi: 10.3390/ijms23169390 (PMC9409473; doi:10.3390/ijms23169390)
Supplement: Supplementary file 1 [file ijms-23-09390-s001.zip › ijms-1843124-supplementary.pdf]

Forsythiaside A Improves the Inhibitory Efficiency of Recombinant Protein Vaccines against Bovine Viral Diarrhea Virus Infection

Guanghui Yang [1], Jiufeng Wang [1], Shenghua Wang [2,*] and Yaohong Zhu [1,*]

[1] College of Veterinary Medicine, China Agricultural University, Beijing 100193, China
[2] OIE Porcine Reproductive and Respiratory Syndrome Reference Laboratory, China Animal Disease Control Center, Beijing 102629, China
* Correspondence: by20173050440@cau.edu.cn (S.W.); zhu_yaohong@hotmail.com (Y.Z.)

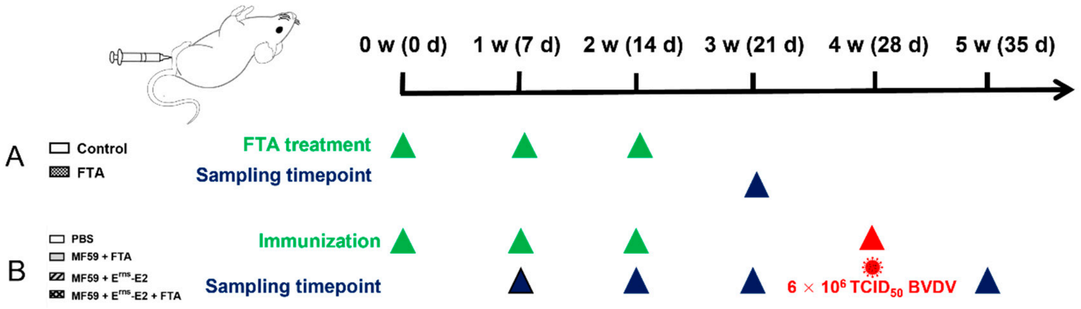

Figure S1. Animal design of treatment and sampling selection process in vivo. (**A**) Twenty of mice were randomly divided into two groups to evaluate the effects of FTA on various organs and T Cell-mediated immune responses in mice. The mice in group I and II were treated intraperitoneally with PBS and FTA at 0, 7, and 14 days (d). (**B**) Eighty female BALB/c mice were divided into four groups (n = 20 per group) to explore the role of the recombinant E^{rns}-E2 protein vaccine-FTA adjuvant combination treatment in preventing BVDV infection mice. Each group was immunized three times intraperitoneally with PBS or MF59 adjuvant and FTA or E^{rns}-E2 protein containing MF59 adjuvant or E^{rns}-E2 protein containing MF59 adjuvant and FTA. Four weeks (W) after the primary immunization, mice were challenged intraperitoneally with 6×10^6 $TCID_{50}$ BVDV. The process of treatment and sample collection are shown in Figure S1.
